# Therapeutic Options for Coronavirus Disease 2019 (COVID-19) - Modulation of Type I Interferon Response as a Promising Strategy?

**DOI:** 10.3389/fpubh.2020.00185

**Published:** 2020-05-15

**Authors:** Aurélien Mary, Lucie Hénaut, Jean-Luc Schmit, Jean-Philippe Lanoix, Michel Brazier

**Affiliations:** ^1^Clinical Critical Care Pharmacy Department, Amiens-Picardie University Hospital, Amiens, France; ^2^UR UPJV 7517, MP3CV, CURS, University of Picardie Jules Verne, Amiens, France; ^3^Infectious Diseases Department, Amiens-Picardie University Hospital, Amiens, France; ^4^AGIR UR UPJV 4294, CURS, University of Picardie Jules Verne, Amiens, France; ^5^Department of Biochemistry, Amiens-Picardie University Hospital, Amiens, France

**Keywords:** COVID-19, SARS-CoV-2, type I interferon, azithromycin, hydroxychloroquine

## Introduction

Since December 2019, China has been experiencing an outbreak of COVID-19, caused by severe acute respiratory syndrome coronavirus 2 (SARS-CoV-2). This disease has spread rapidly to multiple countries and become responsible for the greatest pandemic of the century, as declared by the WHO. The clinical spectrum encompasses asymptomatic infection, mild upper respiratory tract illness, and severe viral pneumonia with respiratory failure (1), with a high mortality estimated around 2% in diagnosed patients (2). Identification of an efficient therapy has now become a major health emergency to avoid health system saturation, especially the medical capabilities of respiratory resuscitation. A large number of clinical trials is currently underway, but no treatment of proven efficacy is known at present. The lack of data from large-scale clinical trials involving COVID-19 patients hampers reliable statistical analyses. In this context of emergency, an in-depth analysis of published preliminary data may help to improve our understanding of disease outcomes and shed light upon potentially efficacious treatment strategies.

## The Initial Focus on Hydroxychloroquine

Since the discovery that the anti-malarial drug hydroxychloroquine (h-CQ) efficiently inhibits SARS-CoV-2 infection *in vitro* (3), numerous clinical studies have been undertaken to test its safety and efficacy in the treatment of COVID-19 associated pneumonia (4). Although preliminary observations from China claimed h-CQ benefit (4), two subsequent publications reported contradictory results on its ability to reduce viral carriage (5, 6).

## Hydroxychloroquine in China

In Shanghai, Chen et al. evaluated the efficacy of h-CQ (400 mg daily for 5 days) in the treatment of 30 symptomatic Chinese patients with COVID-19 (5). They demonstrated that the viral carriage of nasopharyngeal samples (evaluated by PCR at day 7 post-inclusion) was not statistically different between h-CQ and control groups at the end of the follow up. Given that the median duration of viral shedding in COVID-19 has been reported to be around 20 days in Chinese patients (1), it is interesting to note that the viral carriage rapidly decreased in both groups after 4 days of treatment, with 90% of patients proved to be SARS-CoV-2 negative at day 7. Interestingly, all patients included in this study also received some therapies recommended by the National Health Commission (NHC) of China, in particular 100% of them received inhalation of the antiviral cytokine interferon (IFN)-α-2b.

## Type I Interferon Nebulization in the treatment of COVID-19 patients

Type I IFNs (including IFN-α and IFN-β) are antiviral cytokines produced by bronchial epithelial cells in response to viral infection. They display the ability to bind the surface of infected and neighboring cells and promote the induction of around 300 different IFN-inducible genes (ISGs) that subsequently prevent virus protein trafficking, virus RNA synthesis or virion assembly and release (7).

*In vitro*, type I IFN inhibits the replication of both SARS-CoV (8) and SARS-CoV-2 (9) (pre-printed publication). Therefore, the rapid decrease of SARS-CoV-2 carriage observed in the patients of the Chen et al. study may be linked to atomized IFN-α-2b therapy (5). In line with this hypothesis, Liu et al. claimed that a combination therapy of low-dose systematic corticosteroids, lopinavir/ritonavir, and atomization inhalation of IFN-α-2b participated to the observed 0% mortality in their COVID-19 patients in Shenzen, China (10). According to Chen et al., the use of inhaled IFN is also associated with decreased mortality in COVID-19 patients from Wuhan (OR = 2,32 IC95% [1,36;3,97] calculated by our group using the Miettinen method) (11). More recently, Maiti et al. suggested that a polymorphism in the gene encoding IFIH1 (InterFeron-Induced Helicase 1), a host protein that senses the presence of viral RNA and subsequently promotes IFN production, may render African-American more vulnerable to SARS-CoV-2 infection. This observation lead the author to suggest that type I IFN supplement could be developed as an effective treatment for SARS-CoV-2 (12).

Atomized IFN-α-2b is currently the first treatment cited by the Chinese NHC for COVID-19 (13). Indeed, clinical nebulization of IFN-α has been historically used in China to treat viral pneumonia associated with SARS-CoV, middle east respiratory syndrome coronavirus (MERS) and respiratory syncytial virus (14, 15). Its efficiency in treating severe bronchiolitis appears to be superior to the parenteral route^1^ and to expose to fewer undesirable effects, including hematological toxicity, fever, and depression (15).

## Hydroxychloroquine in France

H-CQ was also evaluated in France, and the conclusion of the authors differed from the Chinese study. On March 20th of 2020, Gautret et al. undertook an open label, non-randomized clinical trial in 42 patients with confirmed COVID-19, aimed to evaluate the effect of h-CQ (200 mg TID for 10 days) on SARS-CoV-2 respiratory viral load (6). They observed that h-CQ treatment led to a reduction of viral carriage at day 6. However, when excluding asymptomatic patients as well as patients symptomatic for more than 8 days (potentially in remission phase) none of the seven patients in the control group and only two among the 10 patients treated with h-CQ alone became viral carriage negative (NS; *p* = 0.48) at day 6 (statistical sensitivity analysis performed ourselves). In contrast, a subgroup of six patients treated with both h-CQ and azithromycin (500 mg on day 1, followed by 250 mg daily for the subsequent 4 days) converted to viral carriage negative status at day 6. In line with this observation, a subsequent set of data published by Gautret et al. on March 27th of 2020 showed a rapid fall of nasopharyngeal viral load in 80 patients (5 asymptomatic) treated exclusively with a combination of h-CQ and azithromycin, with 74% of patients becoming SARS-CoV-2 negative at day 6 and 83% negative at day 7 (16).

## Azithromycin in the Treatment of COVID-19 Patients

Apart from its anti-bacterial role, azithromycin has been reported to increase rhinovirus-induced type I and type III IFN response in bronchial epithelial cells from healthy donors (17) asthmatic individuals (18) and patients with chronic obstructive pulmonary disease (19). Azithromycin amelioration of viral-induced IFN also protects against Zika virus infection (20) and reduces the recurrence of severe lower respiratory tract illnesses in children (21). In the study by Gautret et al. (6, 16), the potential anti-viral role of azithromycin was mentioned but not discussed in detail since they mainly focused on h-CQ. However, the possibility must be considered that azithromycin may be responsible for the rapid reduction of viral carriage in this subgroup of h-CQ-treated French patients.

## Anti-Inflammatory Properties of Both Atomized Type I IFN and Azithromycin

Beyond the viral infection, accumulating evidence suggests that a subgroup of patients with severe COVID-19 develop a severe inflammatory syndrome (associated with a dramatical rise in type II IFN and IL-6 serum levels), enhancing disease severity and mortality (22). In this condition both atomized type I IFN and azithromycin may be beneficial as they can also downregulate inflammation (7, 23) and in particular type II IFN pathway *in vitro* (18, 24).

## Discussion

These very recent preliminary data suggest a potential therapeutic benefit of type I IFN pathway stimulation, which may become a key approach in treating COVID-19, possibly in association with direct antiviral agents. The currently ongoing Solidarity and Discovery clinical trials, both of which include an arm of patients treated with IFN-β-1a administered subcutaneously in combination with ritonavir and lopinavir, will help to explore this hypothesis (25). In comparison with this strategy, atomized IFN, which has already shown benefit in China, has the advantage to directly target the lungs and to reduce the risks of systemic side effects. However, it might raise concerns about nebulization side effects, including preservative toxicity, pharmacokinetics, and potential viral dispersion. For these reasons, we suggest that pulmonary nebulization of type I IFN may be useful for patients with a moderate to severe form of the disease and that azithromycin may represent an interesting strategy for patients with less aggressive forms. In favor of this strategy, azithromycin presents the advantage of being cheap, easily applicable to outpatient care and raising less safety concerns than h-CQ, in particular cardiovascular complications. Regarding drug safety, it has to be underlined that under specific conditions type I IFN response can increase the susceptibility to bacterial assault (26, 27). In particular, bacterial infections have been noted in patients receiving prolonged systemic IFN-α-2b therapy for chronic hepatitis C virus infection (28–30). Since preliminary data obtained in China suggest that patients under type I IFN therapy may rapidly become viral carriage negative, a close monitoring of the viral load may be useful to limit treatment duration and subsequent bacterial infection. In this context, the use of azithromycin for outpatient care may even be more favorable than the use of type I IFN itself, given that its anti-bacterial properties may also prevent secondary infections that can occur in association with COVID-19. Nevertheless, the risk of antibiotic resistance linked to an excessive use of azithromycin should not be neglected. Further researches are clearly needed to examine these hypotheses.

## Author Contributions

The authors shared their multidisciplinary skills on the analysis of the best possible therapeutic options to treat COVID-19, based on published or pre-printed data. AM, LH, and MB developed the initial hypotheses and wrote the manuscript. J-PL and J-LS validated the proposed therapeutic approach and critically reviewed the manuscript.

## Conflict of Interest

AM declares travel and accommodation support from Pfizer. J-PL declares financial support from Sanofi, MSD, Pfizer, Gilead, Janssen-Cilag, Mylan, Eumedica. J-LS declares financial support from Astellas, MSD, Abbvie, Astellas, Overcome, Mylan and Mundipharma. These funders had no role in study design, data collection and analysis, decision to publish, or preparation of the manuscript. The remaining authors declare that the research was conducted in the absence of any commercial or financial relationships that could be construed as a potential conflict of interest.

## Footnotes

^1^http://en.cnki.com.cn/Article_en/CJFDTotal-EKYX200305019.htm
